# Viral etiology among children hospitalized for acute respiratory tract infections and its association with meteorological factors and air pollutants: a time-series study (2014–2017) in Macao

**DOI:** 10.1186/s12879-022-07585-y

**Published:** 2022-07-03

**Authors:** Cheng Lei, Cheong Tat Lou, King Io, Kin Ian SiTou, Chong Pak Ip, HongJin U, Baoquan Pan, Carolina Oi Lam Ung

**Affiliations:** 1 Department of Pediatrics, Kiang Wu Hospital, Macao, China; 2 State Key Laboratory of Quality Research in Chinese Medicine, Institute of Chinese Medical Science, University of Macau, Macao, China; 3 Department of Public Health and Medicinal Administration, Faculty of Health Sciences, University of Macau, Macao, China

**Keywords:** Acute respiratory infections, Meteorological factors, Air pollutants, Children, Hospitalization

## Abstract

**Background:**

The associations between viral etiology of acute respiratory infections (ARI) with meteorological factors and air pollutants among children is not fully understood. This study aimed to explore the viral etiology among children hospitalized for ARI and the association of meteorological factors and air pollutants with children hospitalization due to viral ARI.

**Methods:**

Electronic health record data about children (aged between 1 month and 14 years) admitted for ARI at Kiang Wu Hospital in Macao between 2014 and 2017 was analyzed retrospectively. xMAP multiplex assays were used to detect viruses in the nasopharyngeal swab and distributed-lag nonlinear model (DLNM) was used to evaluate associations.

**Results:**

Among the 4880 cases of children hospitalization due to ARI, 3767 (77.2%) were tested positive for at least one virus and 676 (18%) exhibited multiple infections. Enterovirus (EV)/rhinovirus (HRV), adenovirus (ADV), respiratory syncytial virus (RSV) and influenza virus (IFV) were the most common viral pathogens associated with ARI and human bocavirus (hBOV) exhibited the highest multiple infection rates. Meteorological factors and air pollutants (PM_10_, PM_2.5_ and NO_2_) were associated with the risk of viral ARI hospitalization. The relative risk of viral infection increased with daily mean temperature but plateaued when temperature exceeded 23 °C, and increased when the relative humidity was < 70% and peaked at 50%. The effect of solar radiation was insignificant. Air pollutants (including PM_10_, PM_2.5,_ NO_2_ and O_3_) showed strong and immediate effect on the incidence of viral infection.

**Conclusions:**

The effects of mean temperature, relative humidity and air pollutants should be taken into account when considering management of ARI among children.

**Supplementary Information:**

The online version contains supplementary material available at 10.1186/s12879-022-07585-y.

## Introduction

Acute respiratory tract infection (ARI) is one of the most common infectious diseases in children. It has been estimated that, regardless the geographic location or economic status, young children experience 3–6 episodes of ARI on average every year [1]. ARI may affect children’s upper respiratory tract (from the nostrils to the vocal cords in the larynx including the sinuses and the middle ear) or lower respiratory tract (from the trachea and bronchi to bronchioles and alveoli). Children with ARI may also be subject to the risks of extended infection, inflammation, and reduced lung function. It remains one of the major causes of pediatric outpatient and inpatient care, hospital referral and admission, mortality, and morbidity in children under 5 years of age.

Viral pathogens are largely responsible for ARI in children. The positive rate of viral infections among ARI in children ranges from 32.3 to 70% [2–6] and can reach 80% or above in upper respiratory tract infection [7, 8]. More than 200 viral serotypes were found to be associated with human respiratory diseases [9] and the most frequently reported virus found in ARI among young children included influenza A (IFV-A), influenza B (IFV-B), respiratory syncytial viruses A and B (RSV), human coronaviruses 229E, OC43, HKU1 and NL63 (hCoV), human metapneumoviruses (hMPV), human parainfluenza virus types 1, 2, 3, and 4 (PIV-1, PIV-2, PIV-3, and PIV-4), human enteroviruses (EV)/human rhinoviruses (HRV), human adenoviruses (ADV), and human bocavirus (hBoV) [10–12]. The prevalence of respiratory virus is region-specific [13–15]. For instance, in China, the most common virus causing ARI in children varied across different areas and was found to be HRV in Chengdu, RSV in Beijing, HRV/EV in Shanghai, and HCoV in Guangzhou [16–19].

Previous studies have shown that the viral etiology of ARI in children is subject to the impact of outdoor environments including meteorological factors (temperature, humidity, solar radiation and wind speed) [20, 21] and air pollutants (such as Particulate Matter < 10 μm (PM_10_), Particulate Matter < 2.5 μm (PM_2.5_), nitric dioxide (NO_2_), and ozone (O_3_)) [22, 23]. However, the results about the effect of meteorological factors and air pollutants on the ARI viral etiology are inconclusive. For instance, while epidemiology studies in China and the US showed that positive associations existed between PM_2.5_ exposure and ARI in children [23, 24], other studies found no association between them [25, 26]. At present, few studies have been conducted on children in the subtropical marine monsoon climate zone. Therefore, this study aimed to explore the viral etiology among children hospitalized for ARI and the association of meteorological factors and air pollutants with children hospitalization due to viral ARI.

## Methods

### Study site

Macao, a territory with only 32.8 km^2^, is located on the subtropical coast of China and has a typical subtropical marine monsoon climate featured with rich heat, high humidity, warm temperature, and heavy seasonal rainfall, featuring an average annual temperature of 22.6 °C, mean relative humidity of 78.8% and total precipitation of 2058.1 mm [27]. The air quality of Macao is heavily influenced by external factors such as human movement and activities in the neighboring Guangdong province [28].

### Data sources and participants

The demographic, epidemiological and clinical information of eligible patients was extracted from the electronic health records of Kiang Wu Hospital—one of the major hospitals in Macao [29]. Inclusion criteria were: (1) inpatients aged between 1 month and 14 years; (2) admitted to Kiang Wu Hospital for ARI between 2014 and 2017; and (3) the patient profiles must have complete results of the nasopharyngeal swab tests. The diagnosis of ARI was made according to the clinical guidelines recommended by the World Health Organization (with at least two of the following: fever, sore throat, cough, rhinorrhea, nasal congestion and hoarseness). Patients with congenital pneumonia (ICD-10 code: P23), nosocomial infections (ICD-10 code: Y95) or chronic tuberculosis (ICD-10 code: A15) were excluded.

### Multiplex polymerase chain reaction tests

The nasopharyngeal swab specimens were collected from the patients at admission using a special swab (ESWAB from Copan Italia SpA) by nursing staff and transported to the laboratory immediately at a storing temperature of 20–25 °C for testing within 1 h of collection. A qualitative nucleic acid multiplex test (The xTAG^®^ Respiratory Viral Panel FAST v2, Luminex) was performed to test the following respiratory viruses: IFV-A, IFV-B, RSV, hCoV, hMPV, PIV-1, PIV-2, PIV-3, and PIV-4, EV/HRV, ADV, and hBoV. Nucleic acid extraction was performed using the EZ1 DSP Virus Kit (Qiagen, Germany).

### Meteorological and air pollutant data

Meteorological and air pollutant data was obtained from the Macao Meteorological and Geophysical Bureau. Meteorological data included daily mean temperature (°C), daily mean relative humidity (%), daily mean solar radiation duration (h), and daily mean wind speed (km/h). Air pollutant data included daily level of PM_10_ (μg/m^3^), PM_2.5_ (μg/m^3^), NO_2_ (ppb) and O_3_ (ppb).

### Statistical analysis

Chi-square test was used for comparisons between groups. Spearman correlation analysis was used to evaluate the associations between the meteorological and air pollution factors. A value of P < 0.05 was considered statistically significant. All the analyses were performed with the Statistical Package for the Social Sciences (SPSS) V23.0. A distributed lag nonlinear model (DLNM) was used to assess the nonlinear and lagged effects of meteorological factors and air pollutants on ARI hospitalizations as demonstrated in Eq. 1 shown in the following:

1 $${\text{LogE}}\left[ {{\text{Yt}}} \right] = {\text{cb}}\left( {{\text{Temp}}} \right) + {\text{cb}}\left( {{\text{RH}}} \right) + {\text{cb}}\left( {\text{Wind speed}} \right) + {\text{cb}}\left( {{\text{solar}}} \right) + {\text{cb}}\left( {{\text{PM}}_{{{1}0}} } \right) + {\text{cb}}\left( {{\text{O}}_{{3}} } \right) + {\text{s}}\left( {{\text{day}}} \right) + \alpha {\text{Dowt}}$$

where Yt refers to the counts of cases occurred on day t; cb refers to the cross-basis function built up using the DLMN() package in R, combining functions for both exposure and lag dimensions; a maximum lag of 14 days was applied to reflect the incubation period of 3–10 days for ARI; s() refers to the natural cubic spline function to depict long-term time trend; Dowt refers to the day of the week on day t; α is the coefficient of the corresponding terms.

Sensitivity analyses were conducted by applying different df for the lag terms to the models to observe the robustness of the results. All analyses were performed with the DLNM packages in R software version 3.5.3 (https://www.r-project.org/). The significance level was set at P < 0.05.

### Ethics

The study was approved by the Ethics Committee of Kiang Wu Hospital, Macao (Reference number: 2020-003). Considering that all data was collected retrospectively and anonymized in a standardized case report form in the hospital database, informed consent from the patients was neither feasible nor deemed necessary, and was therefore exempted by the committee.

## Results

As shown in Table 1, there were 4880 children admitted for ARI to Kiang Wu Hospital between Jan 2014 and Dec 2017, of them 2070 cases (55%) were male, 2462 cases (50.5%) were of toddler age, and 2712 cases (55.6%) were admitted in spring or summer.

**Table 1:** Descriptive statistics of ARI admissions

	Total cases (N = 4880)		Negative cases (N = 1113)		Positive cases (N = 3767)		χ^2^	P
	n	%	n	%	n	%		
Sex								
Male	2707	55.5	587	52.7	2120	56.3	4.4	< 0.05
Female	2173	44.5	526	47.3	1647	43.7		
Age group								
Infants (1–11 months)	761	15.6	162	14.6	599	15.9	119.9	< 0.001
Toddlers (1–2 years)	2462	50.5	456	41.0	2006	53.3		
Pre-school (3–5 years)	1263	51.3	325	29.2	938	24.9		
School age (6–14 years)	394	8.1	170	15.3	224	5.9		
Season								
Spring (March, April, May)	1406	28.8	311	27.9	1095	29.1	11.9	< 0.05
Summer (June, July, August)	1306	26.8	362	32.5	994	26.4		
Autumn (September, October, November)	1176	24.1	216	19.4	960	25.5		
Winter (December, January, February)	992	20.3	224	20.1	768	20.4		
Diagnosis								
Upper respiratory tract infection	2460	50.4	655	58.8	1805	47.9	98.784	< 0.001
Croup	141	2.9	18	1.6	123	3.3		
Bronchiolitis	512	39.2	37	3.3	475	12.6		
Pneumonia	1097	22.5	264	23.7	833	22.1		

### Viral etiology

**Fig. 1:**
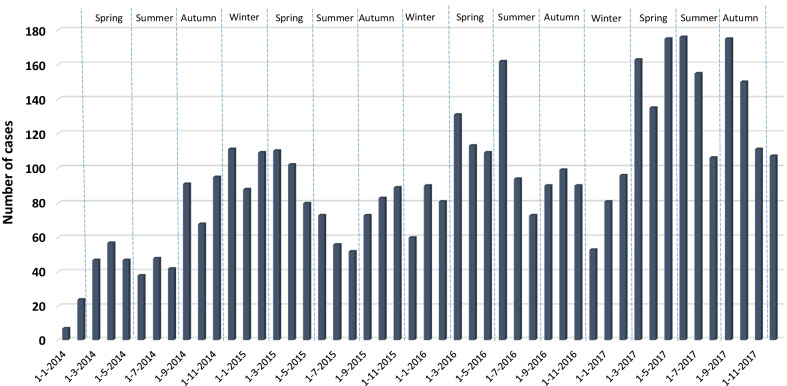
Number of ARI-related admissions tested positive for viral infection during the study period (2014–2017)

Out of the 4880 ARI-related admissions, 3767 cases (77.2%) were tested positive for at least one virus (Table 1). Single infection was detected in 3091 (63.3%) cases. The prevalence of viral infection was higher among male (n = 2120, 56.3%). The toddlers’ group had the highest detection rate and the school-age group had the lowest (χ^2^ = 119.092, P < 0.001). The major diagnosis of viral ARIs included upper respiratory tract infection (n = 1805, 47.9%) and pneumonia (n = 833, 22.1%). Overall, the detection of viral infection peaked in spring (n = 1059, 29.1%) (Fig. 1).

**Fig. 2:**
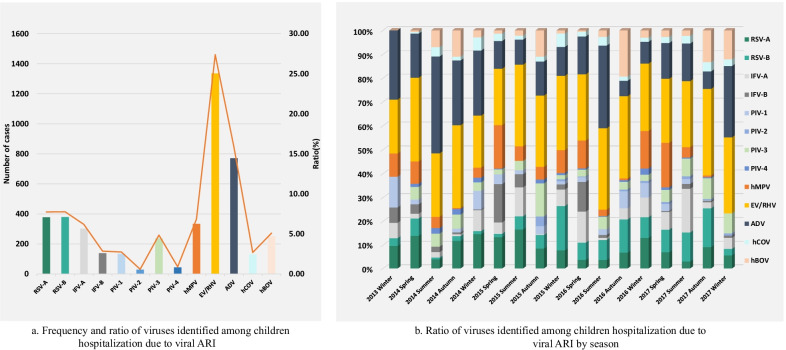
Viral etiology of children hospitalization due to viral ARI

In descending order of detection rate, the most common viruses were EV/HRV, ADV, RSV (RSV-A and RSVB), IFV, PIV, hMPV, hBOV and hCOV (Fig. 2a). On a seasonal basis, EV/HRV, ADV and RSV remained the most common viruses across throughout the study period (Fig. 2b). Proportionally, some viruses were detected more often at times during the study period. For instance, hMPV was less common in autumn than in other seasons while hBOV was more common in autumn than in other seasons.

**Fig. 3:**
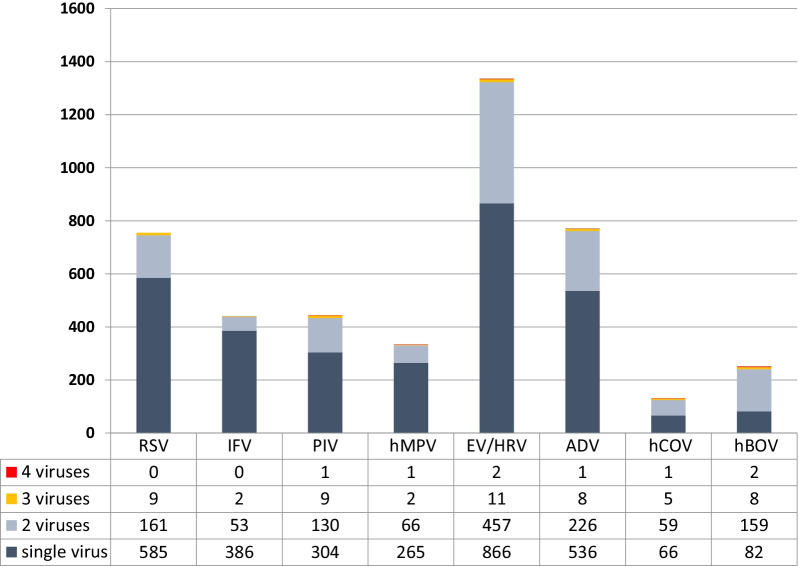
Detection rates of single and multiple viral infections among children hospitalization due to viral ARI

The detection rates of single infection and multiple infections are shown in Fig. 3. hBOV has the highest multiple infection rates (67.3%, 169/251), followed by hCOV (49.6%, 65/131) and EV/HRV (35.2%, 470/1336).The most frequent combination of multiple infections is hBOV and EV/HRV (32.7%, 82/251).

### Meteorological factors and air pollutants

The official metrological and air pollutant data dated between 2014 and 2017 (including temperature, humidity, solar radiation, wind speed, and the air pollutants) is presented as the average value per unit, and the average of the 5th, 25th, 75th and 95th percentiles as shown in Table 2.

**Table 2:** Descriptive statistics of meteorological and air pollution factors

	Positive case (n/day)	Temperature (°C)	Humidity (%)	Solar radiation (h/day)	Wind speed (km/h)	O_3_ (ppb)	NO_2_ (ppb)	PM_2.5_ (ug/m^3^)	PM_10_ (ug/m^3^)
Average	3.34	22.92	82.31	4.7	10.6	26.71	29.59	31.76	58.38
SD	3.08	5.54	10.68	4.05	4.41	15.52	12.24	23.61	31.44
Min	0	3.6	35	0	2	0.05	4.34	0	7.83
P5	0	13.3	62	0	6	6.88	12.63	1.83	19.33
P25	1	18.2	78	0.2	7	15.38	19.97	13.61	34.88
P50	3	24.3	84	4.5	10	22.71	28.76	27.08	52.17
P75	5	27.9	90	8.7	12	36.25	37.62	45.17	75.86
P95	9	29.5	96	10.9	19	54.9	51.31	74.87	116.8
Max	19	32.6	100	12.7	36	98.02	77.82	158.79	243.83

The association of meteorological factors and air pollutants with the incidence of viral infection among children hospitalized for ARI.

Using spearman analysis, the linear relationship between virus infection and the metrological factors was not significant and the correlations identified were only weak positive or weak negative (Additional file 1: Appendix 1). Considering the linear analysis could not reflect the impact of changes in weather and air factors on the risk of virus infection, DLNM was, therefore, employed to analyze the association of meteorological and air pollutant factors with children hospitalization due to viral ARI. With DLNM, our study showed a nonlinear relationship and lagged effect between daily meteorological variables and viral infection incidence.

#### The effect of temperature change on the incidence of viral infection among children hospitalized for ARI

**Fig. 4:**
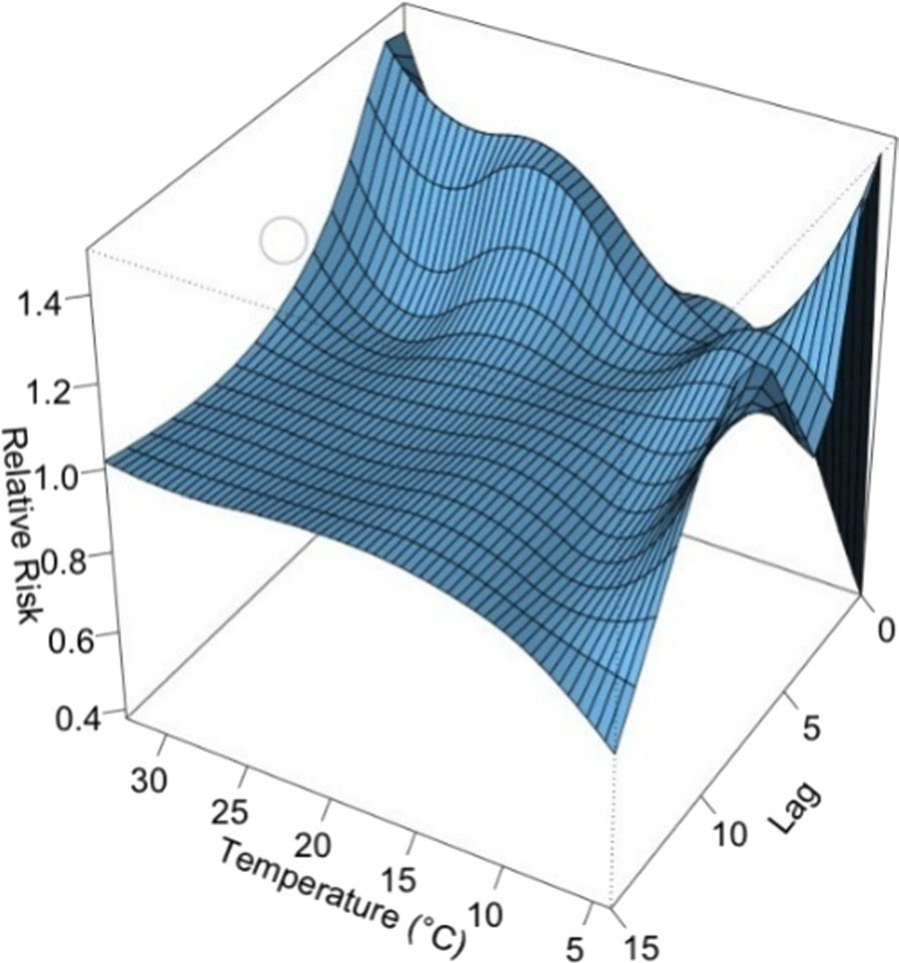
A 3D plot of RR along with temperature and lags

Daily mean temperature had the most significant influence. In Fig. 4, the overall effect of temperature is shown with a 3-D graph of the relative risk (RR) along with temperature and lags compared with a reference value of 10 °C, the point of overall minimum incidence.

**Fig. 5:**
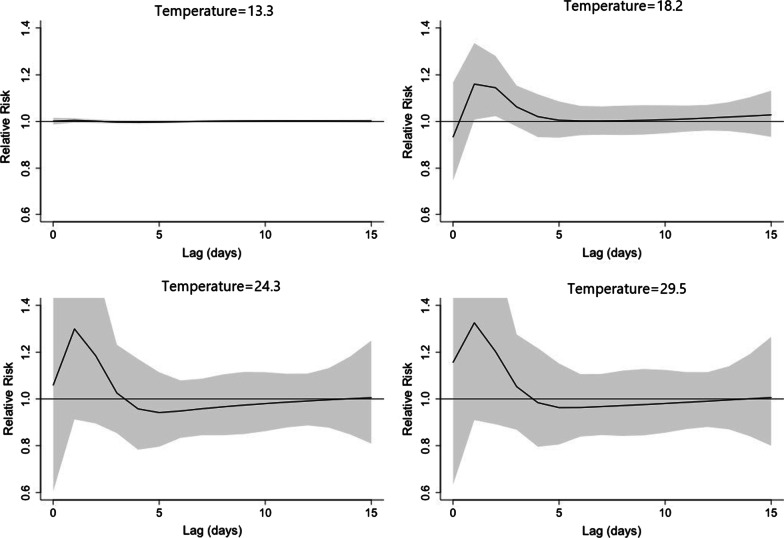
A plot of RR by temperature at 5th, 25th, 75th and 95th percentiles of temperature distribution

**Fig. 6:**
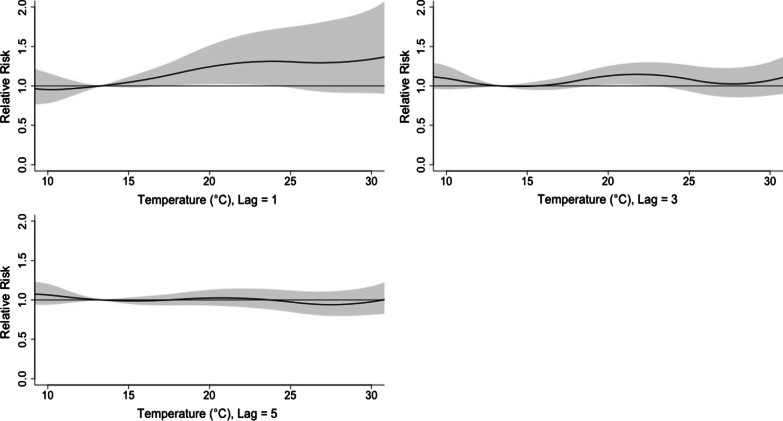
A plot of RR by temperature at specific lags

Figure 5 showed the RR by lag at 4 specific temperatures (13.3, 18.2, 24.3, 29.5 °C), corresponding approximately to 5th, 25th, 75th and 95th percentiles of temperature distribution. Figure 6 confirmed a delayed effect, which suggested that the effect of short-term changes in temperature on viral infection was 1–5 days. By using the appropriate lag time, the association between daily mean temperature and the risk of viral respiratory infection was discovered. The maximum effect of temperature was reached on day 1 (Fig. 5). The RR of viral infection incidence increased with the daily mean temperature, and then plateaued for a temperature higher than 23 °C. The lagged effect lasted for about 5 days.

#### The effect of relative humidity on the incidence of viral infection among children hospitalized for ARI

**Fig. 7:**
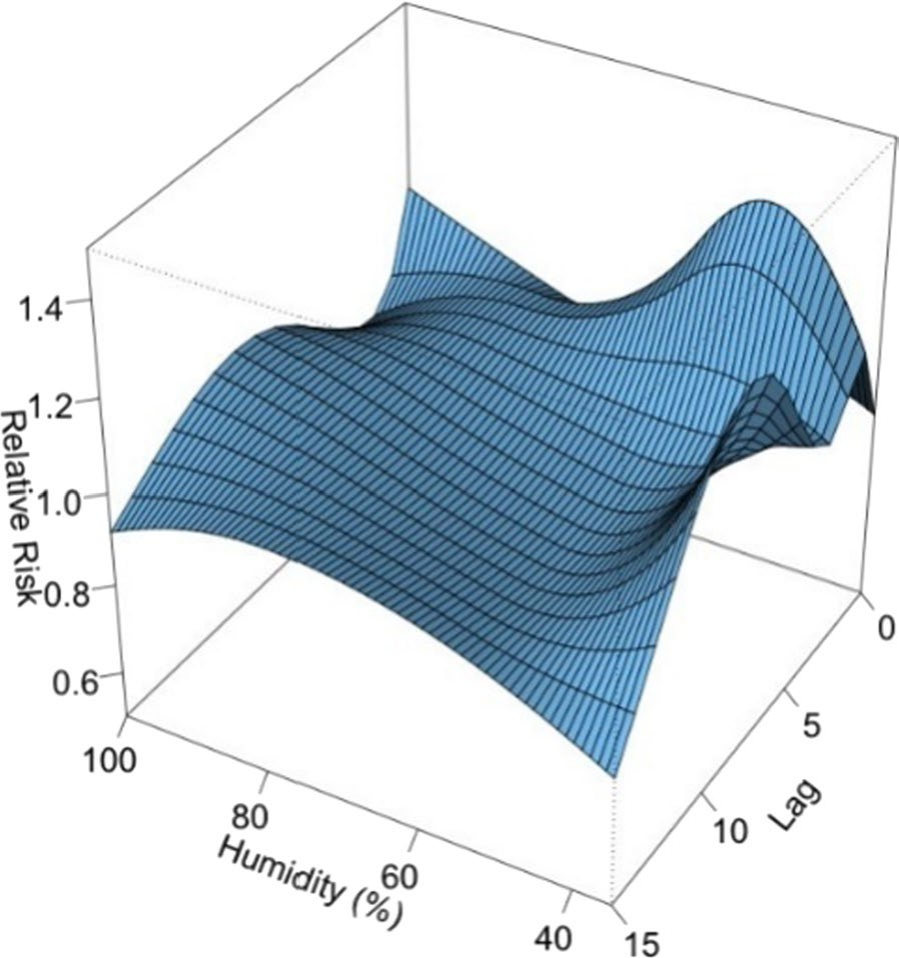
A 3D plot of RR along with humidity and lags

**Fig. 8:**
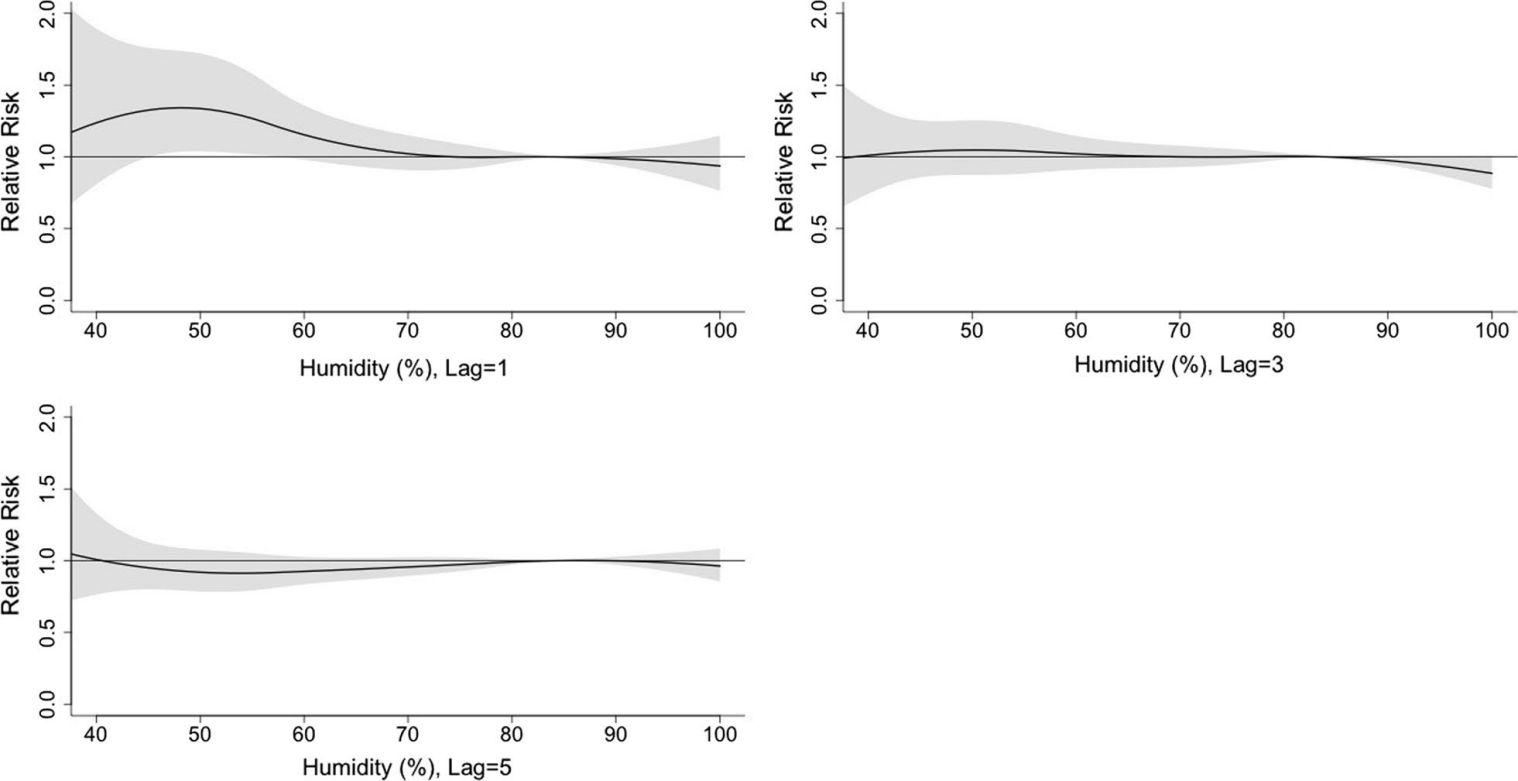
A plot of RR by humidity at specific lags

Figure 7 shows the 3D plot of RR along with humidity and lags. The plot showed a rapid effect of relative humidity on the incidence of viral infection. The RR increased when the relative humidity was < 70% and peaked at 50% (Fig. 8). The lagged effect lasted for about 3 days.

#### The effect of solar radiation on the incidence of viral infection among children hospitalized for ARI

**Fig. 9:**
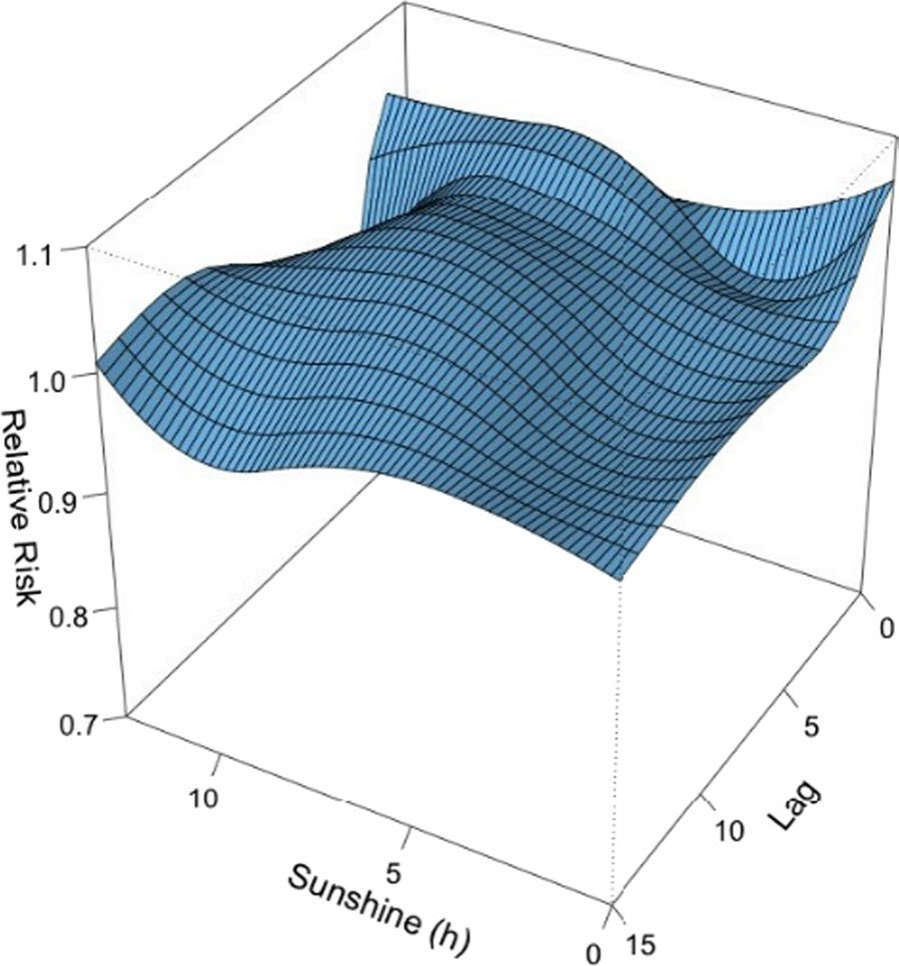
A 3D plot of RR along with solar radiation and lags

**Fig. 10:**
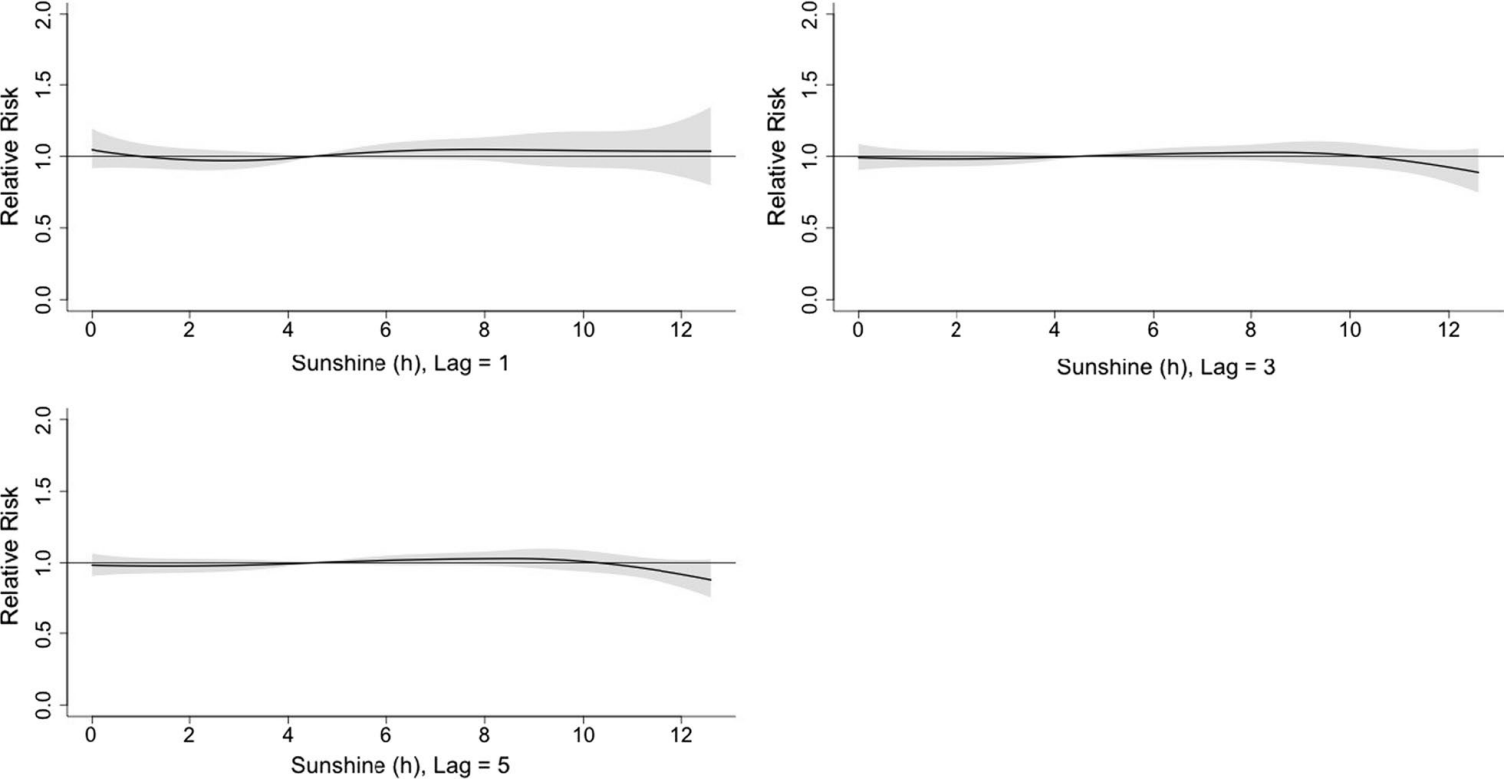
A plot of RR by solar radiation at specific lags

Figure 9 was the 3D plot of RR along with solar radiation and lags. The 3D plot and the plots of different lags showed that the effect of solar radiation on viral activity was insignificant (Fig. 10).

#### The effects of PM_10_, PM_2.5_ and NO_2_ on the incidence of viral infection among children hospitalized for ARI

**Fig. 11:**
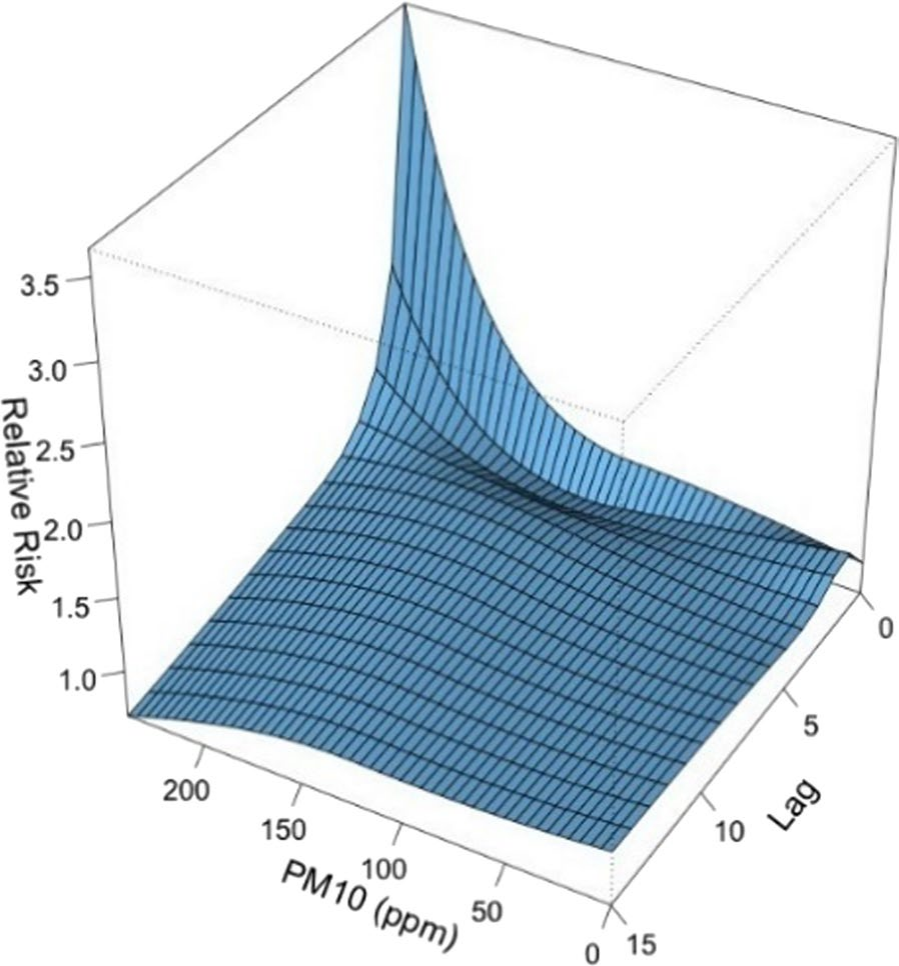
A 3D plot of RR along PM_10_ and lags

**Fig. 12:**
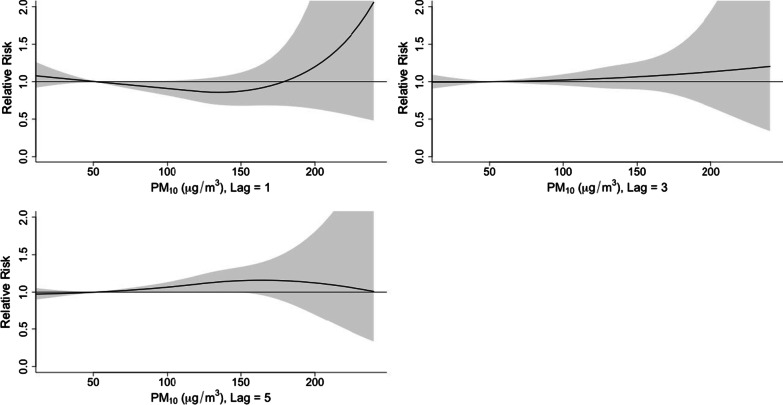
A plot of RR by PM_10_ at specific lags

Since PM_10_, PM_2.5_ and NO_2_ are highly correlated according to the above spearman’s correlation analysis, we used PM_10_ to represent the 3 pollutants in this study. The 3D plot of RR along PM_10_ and lags is provided in Fig. 11. The plot showed a very strong and immediate effect of PM_10_. The RR increased significantly when the PM_10_ level was over 150 ug/m^3^. The lagged effect lasted for about 5 days (Fig. 12).

#### The effects of O_3_ on the incidence of viral infection among children hospitalized for ARI

**Fig. 13:**
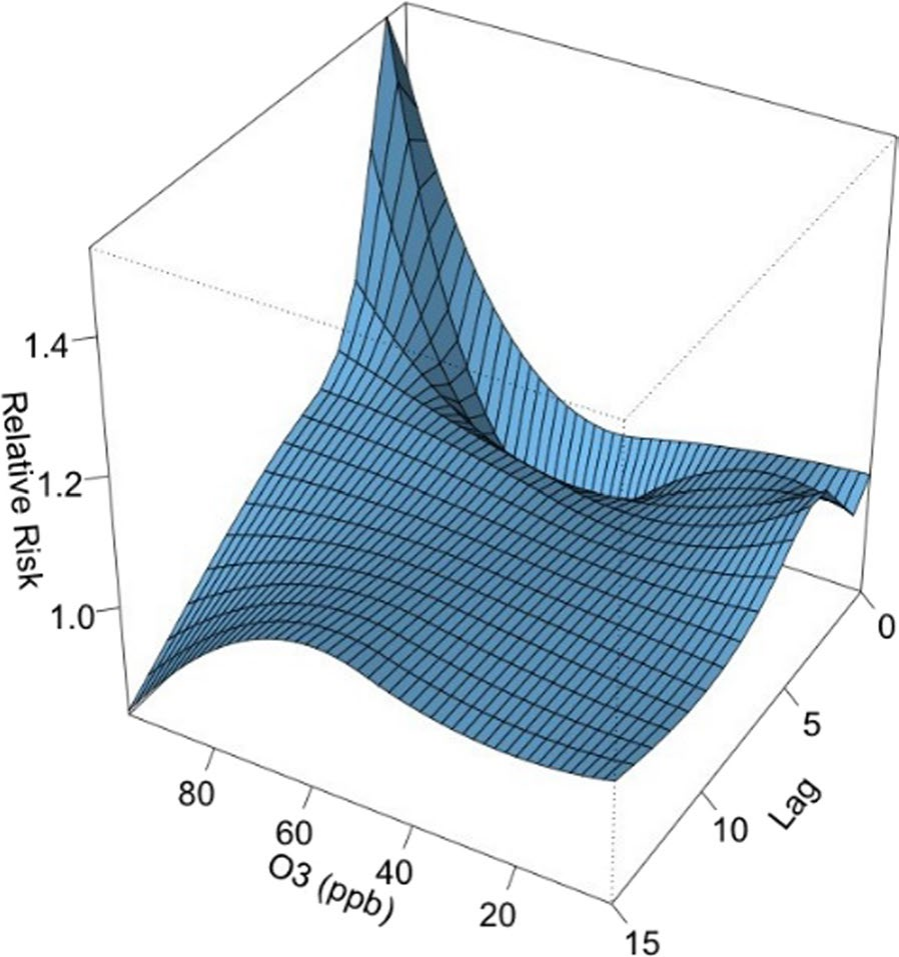
A 3D plot of RR along O_3_ and lags

**Fig. 14:**
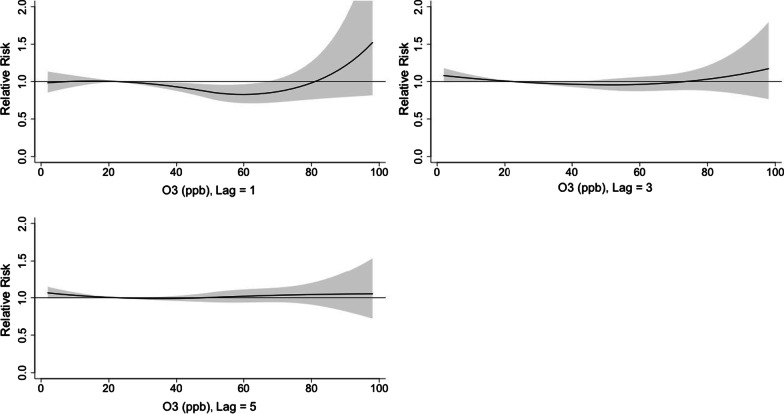
A plot of RR by O_3_ at specific lags

The 3D plot of RR along O_3_ and lags was provided in Fig. 13. The plot showed a very strong and immediate effect of O_3_. The RR increased significantly when the O_3_ level was over 80 ppb. The lagged effect lasted for about 3 days (Fig. 14).

## Discussion

In this study, we found that the detection rate of viral respiratory infection among children hospitalized for ARI was 77.2%, while previous studies showed that about 31.2–86% of respiratory infection was caused by virus [13, 15, 30]. The most prevalent pathogens among admitted children in this study were EV/HRV (1336, 27.4%), ADV (771, 15.8%), and RSV (756, 15.4%). To analyze the association of meteorological and environmental factors with viral etiology, we constructed a DLNM model that revealed the lagged effect of short-term changes in meteorological environmental factors on the incidence of virus infection among children hospitalized due to ARI in Macao. Collectively, the study findings not only provide the health authority with local information to inform health policies, but also supplement the current understanding about the impact of meteorological and environmental factors on the viral etiology of ARI among children.

The high incidence of viral ARI found in the toddler group was similar to the results of the Korean study [13] and was probably related to the immature immune system of younger cases and the high risk of cross-infection in nurseries. Multiple infections were found to be 13.9% (676 cases) which fell within the range 4.9–42% determined previously [13–15, 30, 31]. hBOV had the highest rate of multiple infections, which was also consistent with previous research [32, 33]. The toddler group had the highest multiple infection rates, similar to a Spanish study [34].

Scotta et al. found that multiple viral infection did not influence clinically important outcomes such as length of stay, length of supplemental oxygen, need for hospitalization, supplemental oxygen, need of intensive care, mechanical ventilation and death [35]. Similarly in this study, no statistical significance was found between the multiple infections and the clinical presentation of bronchiolitis, pneumonia or croup. Recent findings suggested that one virus infection could be blocked by the presence of another through different mechanisms, such as resource competition of host cells, immune response, or interference through viral proteins [36]. Therefore, multiple viral infection was not necessarily associated with more severe illness [37].

Meteorological and environmental factors have been shown to have important influence on human health, especially the respiratory system and the relationship between them were not necessarily linear [38]. As found in this study, the effect of meteorological and pollution factors was often delayed in time. This situation occurs frequently when assessing the short-term effects of environmental changes and may cause a harvesting effect: a phenomenon that arises when a stressor affects a specific population. After some periods with excess mortality, however, a decrease in overall mortality during the subsequent periods had also been observed [39]. Therefore, linear analysis and correlation analysis may not be very useful for reflecting the effects of meteorological factors on respiratory diseases. Recently, the distributed non-linear model has been widely used to describe the non-linear relationship and delayed effect between meteorological factors and health outcomes [40, 41].

In this study, the daily mean temperature, relative humidity, and air pollutants demonstrated important influence on the incidence of viral respiratory infection in children, while the effect of solar radiation was found to be insignificant. The effect of temperature was most significant on day 0–5 and peaked on day 1. The RR increased with the daily mean temperature, and then plateaued for a temperature higher than 23 °C. A rapid effect of relative humidity on viral activity was also noted in which the RR increased when the relative humidity was < 70% and peaked at 50%. This suggests that low humidity can be a risk factor for viral respiratory infection in children. However, the effect of solar radiation on the risk of viral infection incidence is insignificant.

High air pollutant level has been associated with increased asthma attacks and emergency department visits caused by respiratory diseases [42–44]. In recent years, studies of viral respiratory infection among children in different regions had been published [45, 46]. Since the environment varied in different regions, for the first time we tried to explore the association between meteorological factors and viral infection among children hospitalized for ARI in Macao. Air pollutants were known to have great influence on the risk of viral respiratory infection among children. In Macao, the levels of PM_10_, PM_2.5_ and NO_2_ were positive correlated. Elevated level of air pollutants can be harmful to children’s respiratory tracts and cause increased risk of viral infection [23, 45–47]. Fernando et al. described that fine particulate matter can cause inflammation of airway mucosa and airway clearance dysfunction [48].

Fine particulate matters in human alveolar epithelial cell A549 can induce oxidative stress and inflammation. Oxidative stress was characterized by elevated intracellular levels of reactive oxygen species (ROS) and production of antioxidant enzyme (superoxide dismutase (SOD), catalase (CAT) and hemeoxygenas-1 (HO-1)). During oxidative stress, TNF-α and IL-6 levels increase and cause cell damage. Cell damage is characterized by decline in cell activity, release of LDH increases, formation of apoptotic body and necrosis. All these eventually lead to inflammation of lung tissue and decrease local resistance to infection. Our study confirmed that high levels of air pollutants were associated with an increased risk of viral infection and the lag effect lasted for 3–5 days. Hence, if high level of air pollution was detected, the government could advise the public to avoid unnecessary outdoor activities in the next 5–7 days, in order to reduce the delayed effect on the incidence of respiratory viral infection.

This study has several limitations. Since only a relatively small sample size was included in this study, more regional information and studies are needed to support our conclusion. Second, we were unable to gather individual-level data such as socioeconomic status or secondary smoking status due to the nature of the data. Third, we were not able to control the unmeasured confounding factors. The study findings may only provide a snapshot of the temporal fluctuations in the associated identified. Thus, our results may be at best generalized to the population in Macao.

In order to inform the management of viral ARI among children in the future, further studies may focus on the following areas: (1) as some common viruses had been identified, the association of meteorological factors and air pollutants with specific virus responsible for viral ARI may be assessed; (2) the severity of the symptoms caused by viral ARI among hospitalized children should also be taken into account when analyzing the viral etiology and its associations with meteorological factors and air pollutants; (3) similar analysis of the associations may be conducted by season to determine if there are any seasonal variations; and (4) the effect of meteorological factors and air pollutants on the symptoms experienced by children hospitalized for viral ARI.

## Conclusion

In conclusion, EV/HRV, ADV, RSV and IFV are common viral pathogens of respiratory infection among children hospitalized for ARI in Macao. Multiple infections of viruses are very common and hBOV has the highest multiple infection rate. Daily mean temperature, relative humidity and air pollutants (PM_10_, PM_2.5_, NO_2_ and O_3_) have significant effect on the risk of viral respiratory infection. The risk of viral infection increases with the level of air pollutants. As the impact of meteorological factors and air pollutants on the viral etiology of ARI in children is region-specific, the findings of this study have important implications for public health policy to mitigate the risk of viral infections under certain environmental influences.

## Supplementary Information

**Additional file 1: Appendix 1.** Spearman correlation analysis of respiratory viruses, meteorological and air pollution factors.

## Data Availability

All data generated or analyzed during this study are included in this published article. The raw data are available from the corresponding author on reasonable request.

## References

[1] Simoes EAF, Cherian T, Chow J, Jamison DT, Breman JG (2006). Acute respiratory infections in children. Disease control priorities in developing countries.

[2] Baroudy NRE, Refay ASE, Hamid TAA (2018). Respiratory viruses and atypical bacteria co-infection in children with acute respiratory infection. Open Access Maced J Med Sci.

[3] Wishaupt JO, van der Ploeg T, de Groot R (2017). Single-and multiple viral respiratory infections in children: disease and management cannot be related to a specific pathogen. BMC Infect Dis.

[4] Tang LF, Wang TL, Tang HF (2008). Viral pathogens of acute lower respiratory tract infection in China. Indian Pediatr.

[5] Yen C-Y, Wu W-T, Chang C-Y (2019). Viral etiologies of acute respiratory tract infections among hospitalized children—a comparison between single and multiple viral infections. J Microbiol Immunol Infect.

[6] Feng L, Li Z, Zhao S (2014). Viral etiologies of hospitalized acute lower respiratory infection patients in China, 2009–2013. PLoS ONE.

[7] Cui B, Zhang D, Pan H (2015). Viral aetiology of acute respiratory infections among children and associated meteorological factors in southern China. BMC Infect Dis.

[8] Zhang G, Hu Y, Wang H, et al. High incidence of multiple viral infections identified in upper respiratory tract infected children under three years of age in Shanghai, China. 2012. 10.1371/journal.pone.0044568 PMC3436764 22970251

[9] Lennette EH (1985). Manual of clinical microbiology.

[10] Selwyn B (1990). The epidemiology of acute respiratory tract infection in young children: comparison of findings from several developing countries. Rev Infect Dis.

[11] De Conto F, Conversano F, Medici MC (2019). Epidemiology of human respiratory viruses in children with acute respiratory tract infection in a 3-year hospital-based survey in Northern Italy. Diagn Microbiol Infect Dis.

[12] Lei C, Yang L, Lou CT (2021). Viral etiology and epidemiology of pediatric patients hospitalized for acute respiratory tract infections in Macao: a retrospective study from 2014 to 2017. BMC Infect Dis.

[13] Choi E, Ha K-S, Song DJ (2018). Clinical and laboratory profiles of hospitalized children with acute respiratory virus infection. Korean J Pediatr.

[14] Li J, Tao Y, Tang M (2018). Rapid detection of respiratory organisms with the FilmArray respiratory panel in a large children’s hospital in China. BMC Infect Dis.

[15] Kurskaya O, Ryabichenko T, Leonova N (2018). Viral etiology of acute respiratory infections in hospitalized children in Novosibirsk City, Russia (2013–2017). PLoS ONE.

[16] Yu J, Xie Z, Zhang T (2018). Comparison of the prevalence of respiratory viruses in patients with acute respiratory infections at different hospital settings in North China, 2012–2015. BMC Infect Dis.

[17] Zhao Y, Lu R, Shen J (2019). Comparison of viral and epidemiological profiles of hospitalized children with severe acute respiratory infection in Beijing and Shanghai, China. BMC Infect Dis.

[18] Chen J, Hu P, Zhou T (2018). Epidemiology and clinical characteristics of acute respiratory tract infections among hospitalized infants and young children in Chengdu, West China, 2009–2014. BMC Pediatr.

[19] Zeng ZQ, Chen DH, Tan WP (2018). Epidemiology and clinical characteristics of human coronaviruses OC43, 229E, NL63, and HKU1: a study of hospitalized children with acute respiratory tract infection in Guangzhou, China. Eur J Clin Microbiol Infect Dis.

[20] Meerhoff TJ, Paget JW, Kimpen JL (2009). Variation of respiratory syncytial virus and the relation with meteorological factors in different winter seasons. Pediatr Infect Dis J.

[21] Yusuf S, Piedimonte G, Auais A (2007). The relationship of meteorological conditions to the epidemic activity of respiratory syncytial virus. Epidemiol Infect.

[22] Karr CJ, Rudra CB, Miller KA (2009). Infant exposure to fine particulate matter and traffic and risk of hospitalization for RSV bronchiolitis in a region with lower ambient air pollution. Environ Res.

[23] Horne BD, Joy EA, Hofmann MG (2018). Short-term elevation of fine particulate matter air pollution and acute lower respiratory infection. Am J Respir Crit Care Med.

[24] Zheng PW, Wang JB, Zhang ZY (2017). Air pollution and hospital visits for acute upper and lower respiratory infections among children in Ningbo, China: a time-series analysis. Environ Sci Pollut Res Int.

[25] Darrow LA, Klein M, Flanders WD (2014). Air pollution and acute respiratory infections among children 0–4 years of age: an 18-year time-series study. Am J Epidemiol.

[26] Karr C, Lumley T, Shepherd K (2006). A case-crossover study of wintertime ambient air pollution and infant bronchiolitis. Environ Health Perspect.

[27] Macao Meteorological and Geophysical Bureau. Weather and climate 2021. https://www.smg.gov.mo/en. Accessed 24 Sept 2021.

[28] Lei MT, Monjardino J, Mendes L (2020). Statistical forecast of pollution episodes in Macao during National Holiday and COVID-19. Int J Environ Res Public Health.

[29] Statistics and Census Service. Healthcare. In: Service. SaC, ed. 2020.

[30] Richter J, Panayiotou C, Tryfonos C (2016). Aetiology of acute respiratory tract infections in hospitalised children in cyprus. PLoS ONE.

[31] Razanajatovo NH, Richard V, Hoffmann J (2011). Viral etiology of influenza-like illnesses in Antananarivo, Madagascar, July 2008 to June 2009. PLoS ONE.

[32] Adam K, Pangesti KNA, Setiawaty V (2017). Multiple viral infection detected from influenza-like illness cases in Indonesia. BioMed Res Int.

[33] Schildgen O (2013). Human bocavirus: lessons learned to date. Pathogens.

[34] Cebey-López M, Herberg J, Pardo-Seco J (2015). Viral co-infections in pediatric patients hospitalized with lower tract acute respiratory infections. PLoS ONE.

[35] Scotta MC, Chakr VCBG, de Moura A (2016). Respiratory viral coinfection and disease severity in children: a systematic review and meta-analysis. J Clin Virol.

[36] Pinky L, Dobrovolny HM (2016). Coinfections of the respiratory tract: viral competition for resources. PLoS ONE.

[37] Asner SA, Science ME, Tran D (2014). Clinical disease severity of respiratory viral co-infection versus single viral infection: a systematic review and meta-analysis. PLoS ONE.

[38] Zhang Y, Li C, Feng R (2016). The short-term effect of ambient temperature on mortality in Wuhan, China: a time-series study using a distributed lag non-linear model. Int J Environ Res Public Health.

[39] Gasparrini A, Armstrong B, Kenward MG (2010). Distributed lag non-linear models. Stat Med.

[40] Gasparrini A (2014). Modeling exposure-lag-response associations with distributed lag non-linear models. Stat Med.

[41] Taj T, Jakobsson K, Stroh E (2016). Air pollution is associated with primary health care visits for asthma in Sweden: a case-crossover design with a distributed lag non-linear model. Spat Spatiotemporal Epidemiol.

[42] Pawankar R, Wang J-Y, Wang IJ (2020). Asia Pacific Association of Allergy Asthma and Clinical Immunology White Paper 2020 on climate change, air pollution, and biodiversity in Asia-Pacific and impact on allergic diseases. Asia Pac Allergy.

[43] Rosenquist NA, Metcalf WJ, Ryu SY (2020). Acute associations between PM25 and ozone concentrations and asthma exacerbations among patients with and without allergic comorbidities. J Expo Sci Environ Epidemiol.

[44] Nhung NTT, Amini H, Schindler C (2017). Short-term association between ambient air pollution and pneumonia in children: a systematic review and meta-analysis of time-series and case-crossover studies. Environ Pollut.

[45] Li Y, Xiao C, Li J (2018). Association between air pollution and upper respiratory tract infection in hospital outpatients aged 0–14 years in Hefei, China: a time series study. Public Health.

[46] Xu Z, Hu W, Williams G (2013). Air pollution, temperature and pediatric influenza in Brisbane Australia. Environ Int.

[47] Xu Q, Li X, Wang S (2016). Fine particulate air pollution and hospital emergency room visits for respiratory disease in urban areas in Beijing, China, in 2013. PLoS ONE.

[48] Fernando IS, Jayawardena TU, Kim H-S (2019). Beijing urban particulate matter-induced injury and inflammation in human lung epithelial cells and the protective effects of fucosterol from Sargassum binderi (Sonder ex J. Agardh). Environ Res.

