# Supplementary material for: Viral etiology among children hospitalized for acute respiratory tract infections and its association with meteorological factors and air pollutants: a time-series study (2014–2017) in Macao
Source: BMC Infect Dis. 2022 Jul 3;22:588. doi: 10.1186/s12879-022-07585-y (PMC9250746; doi:10.1186/s12879-022-07585-y)
Supplement: Supplementary file 1 — Additional file 1: Appendix 1. Spearman correlation analysis of respiratory viruses, meteorological and air pollution factors. [file 12879_2022_7585_MOESM1_ESM.docx]

Appendix 1 Spearman correlation analysis of respiratory viruses, meteorological and air pollution factors.

|  | Temperature | Humidity | Solar radiation | Wind speed | O_3_ | NO_2_ | PM_2.5_ | PM_10_ |
| --- | --- | --- | --- | --- | --- | --- | --- | --- |
| TOTAL | 0.043 | 0.083 | -0.047 | -0.035 | -0.099 | -0.066 | -0.188 | -0.132 |
| RSV-A | -0.062 | 0.026 | -0.064 | 0.005 | -0.009 | 0.038 | 0.033 | 0.04 |
| RSV-B | 0.058 | 0.017 | -0.024 | -0.014 | -0.044 | 0.022 | -0.118 | -0.082 |
| IFV-A | -0.038 | 0.062 | -0.044 | -0.061 | -0.063 | -0.031 | -0.084 | -0.063 |
| IFV-B | -0.017 | 0.188 | -0.116 | -0.055 | -0.144 | -0.122 | -0.037 | -0.067 |
| PIV | 0.003 | -0.006 | -0.012 | 0.009 | 0.017 | 0.038 | -0.049 | -0.019 |
| hMPV | -0.096 | 0.123 | -0.095 | -0.033 | -0.063 | -0.085 | -0.037 | -0.024 |
| EV/HRV | 0.051 | 0.057 | -0.002 | -0.01 | -0.095 | -0.074 | 0.005 | -0.052 |
| ADV | 0.035 | 0.039 | -0.018 | -0.029 | -0.04 | -0.047 | -0.054 | -0.038 |
| hBOV | 0.069 | -0.112 | 0.071 | 0.051 | 0.060 | 0.094 | -0.047 | 0.004 |
| Temperature | 1 | 0.058 | 0.465 | -0.447 | -0.007 | -0.515 | -0.618 | -0.563 |
| Humidity |  | 1 | -0.562 | -0.246 | -0.521 | -0.528 | -0.382 | -0.488 |
| Solar radiation |  |  | 1 | -0.184 | 0.283 | -0.01 | -0.106 | -0.04 |
| Windspeed |  |  |  | 1 | 0.153 | 0.183 | 0.224 | 0.215 |
| O3 |  |  |  |  | 1 | 0.499 | 0.391 | 0.469 |
| NO2 |  |  |  |  |  | 1 | 0.784 | 0.828* |
| PM2.5 |  |  |  |  |  |  | 1 | 0.945* |
| PM10 |  |  |  |  |  |  |  | 1 |

Grey boxes: P<0.05, * significant correlation.
